# Association between bone mineral density and content and physical growth parameters among children and adolescents diagnosed with HIV: a cross-sectional study

**DOI:** 10.1590/1516-3180.2021.0549.R1.03012022

**Published:** 2022-08-01

**Authors:** Suellem Zanlorenci, Priscila Custódio Martins, Carlos Alencar Souza Alves, João Antônio Chula de Castro, Luiz Rodrigo Augustemak de Lima, Edio Luiz Petroski, Diego Augusto Santos Silva

**Affiliations:** I BSc. Master’s Student in Physical Activity and Health, Núcleo de Pesquisa em Cineantropometria e Desempenho Humano (NUCIDH), Universidade Federal de Santa Catarina (UFSC), Florianópolis (SC), Brazil; II MSc. Doctoral Student, Núcleo de Pesquisa em Cineantropometria e Desempenho Humano (NUCIDH), Universidade Federal de Santa Catarina (UFSC), Florianópolis (SC), Brazil.; III MSc. Doctoral Student, Núcleo de Pesquisa em Cineantropometria e Desempenho Humano (NUCIDH), Universidade Federal de Santa Catarina (UFSC), Florianópolis (SC), Brazil.; IV MSc. Doctoral Student, Núcleo de Pesquisa em Cineantropometria e Desempenho Humano (NUCIDH), Universidade Federal de Santa Catarina (UFSC), Florianópolis (SC), Brazil.; V PhD. Adjunct Professor, Instituto de Educação Física e Esporte (IEFE), Universidade Federal de Alagoas (UFAL), Maceió (AL), Brazil.; VI PhD. Associate Professor, Department of Physical Education, Universidade Federal de Santa Catarina (UFSC), Florianópolis (SC), Brazil; and Researcher, Núcleo de Pesquisa em Cineantropometria e Desempenho Humano (NUCIDH), Universidade Federal de Santa Catarina (UFSC), Florianópolis (SC), Brazil.; VII PhD. Associate Professor, Department of Physical Education, Universidade Federal de Santa Catarina (UFSC), Florianópolis (SC), Brazil; and Researcher, Research Center in Kinanthropometry and Human Performance, Florianópolis (SC), Brazil.

**Keywords:** Body composition, HIV, Lifestyle, Growth, Health, Body mass, Antiretroviral therapy, Physical activity, Sedentary behavior

## Abstract

**BACKGROUND:**

During childhood and adolescence, there are significant increases in bone mineral content (BMC) and bone mineral density (BMD).

**OBJECTIVE:**

To investigate physical growth parameters associated with BMD and BMC among children and adolescents diagnosed with human immunodeficiency virus (HIV).

**DESIGN AND SETTING:**

Cross-sectional study conducted in Florianópolis, Brazil, among 63 children and adolescents (aged 8-15 years) diagnosed with HIV.

**METHOD:**

BMD, BMC and fat percentage z score were evaluated using dual X-ray absorptiometry. Age/height z score and body mass index (BMI)/age z score were obtained in accordance with international recommendations, and bone age was obtained through hand-wrist radiography. Sex, family income, information on HIV infection (T CD4+ lymphocyte count, viral load and type of antiretroviral therapy, moderate-vigorous physical activity and sedentary behavior) were used as adjustment variables in the analyses. Simple and multiple linear regression analyses were performed, with a significance level of P ≤ 0.05.

**RESULTS:**

Subtotal BMD (without the head region) was directly associated with bone age, BMI/age z score and fat percentage z score, even after adjusting for covariates. Subtotal BMC/height was directly associated with bone age, height/age z score, BMI/age z score and fat percentage z score, even after adjusting for covariates.

**CONCLUSION:**

Subtotal BMD and subtotal BMC/height were directly associated with physical growth indicators among children and adolescents diagnosed with HIV.

## INTRODUCTION

During childhood and adolescence, significant increases in bone mineral content (BMC) and bone mineral density (BMD) occur in association with the growth spurt and a high mineralization rate.^1^ These factors are considered to be determinants for protection against osteopenia, and later against osteoporosis, during adult life.^2^ Among children and adolescents diagnosed with human immunodeficiency virus (HIV), lower bone mass concentrations and mineralization rates may occur due to impairments of adequate physical growth and consequent pubertal delay associated with HIV infection and prolonged use of antiretroviral therapy (ART).^3,4^

In the growth process, regardless of sex, BMD and BMC concentrations do not develop linearly with chronological age. Their growth rate has been shown to be higher after the growth spurt.^5^ In a study conducted among children and adolescents diagnosed with HIV (aged 8-16 years), it was found that body mass, body mass index (BMI) and body fat were directly associated with BMD.^6^ The mechanisms through which HIV can lead to changes in BMD are not fully understood, but are probably multifactorial, including factors associated with HIV (ART and disease stage)^6^ and modifiable risk factors (changes in physical growth, low physical activity and highly sedentary behavior).^6^ Use of ART can decrease the BMC and BMD of patients diagnosed with HIV.^7^ In a previous study, a direct association between moderate-vigorous physical activity (MVPA) and high gravitational or muscular load and bone mass was identified among children and adolescents without any diagnosis of HIV.^8^ This highlights the importance of factors relating to disease/treatment and lifestyle, which can impact physical growth parameters.

Studies carried out among children and adolescents diagnosed with HIV showed that height/age z score was a bone mass predictor.^6,9^ Cross-sectional studies have only investigated associations between BMD and physical growth parameters, with no data regarding BMC.^6,9,10^ Other studies did not consider lifestyle variables, such as MVPA and sedentary behavior, in correlating BMD and BMC with physical growth parameters.^11,12,13^

## OBJECTIVE

Thus, the aim of this study was to investigate physical growth parameters associated with BMD and BMC among children and adolescents diagnosed with HIV.

## METHODS

### Research characteristics

This was a cross-sectional study that formed part of the “Saúde PositHIVa” study, carried out in the city of Florianópolis, Santa Catarina, Brazil, from August 2015 to June 2016. The study complied with all ethical procedures, and had received approval from the Research Ethics Committee of the Universidade Federal de Santa Catarina (UFSC) (protocol no. 1.410.144) (CAAE protocol: 49691815.0.0000.0121, dated June 2010) and from the Ethics Committee of the “Joana de Gusmão” Children’s Hospital (HIJG) (protocol no. 850.0777, dated January 12, 2009). A free and informed consent statement was signed by the participants’ parents or legal guardians and a consent statement was signed by the participating children and adolescents.

### Population and sample

Children and adolescents diagnosed with HIV (vertical transmission) who were recruited at the outpatient clinic of a regional HIV reference center the HIJG, located in the city of Florianópolis, capital of the state of Santa Catarina, participated in this study. In 2015, among the children and adolescents diagnosed with HIV who were assisted at HIJG, 83 were eligible for this study. However, 14 were excluded because they refused to participate in the research, four withdrew from participation over the course of the study and two did not participate in all the data collection stages necessary for the statistical analyses of the present study. Thus, 63 participants were assessed.

The sample size was calculated *a posteriori* taking into account type I error (α = 0.05) and type II error (β = 0.95), to identify associations between physical growth parameters and BMD and BMC, of medium effect size (0.50). All calculations were performed using the G* Power software, version 3.1.9.2 (Universität Düsseldorf, Germany), and 30% were added for losses and refusals. Thus, for multiple linear regression analysis, the sample of 63 children and adolescents made it possible to find associations with an average effect size of 0.50.

### Eligibility criteria

The inclusion criteria were that the subjects needed to: 1) present HIV infection through vertical transmission in the medical record; 2) be aged 8-15 years; 3) have clinical and laboratory information available in the medical record; and 4) be able to stand and communicate.

The exclusion criteria were the following situations: 1) motor impairment or contraindication of vigorous exercise; 2) speech, hearing and/or cognitive impairment; 3) diseases that change body composition, except for those related to HIV infection; and 4) regular use of diuretic drugs or immunotherapies not related to ART.

### Study variables

#### Dependent variables

BMD and BMC analyses were carried out by means of dual emission X-ray absorptiometry (DXA). DXA measurements were performed at the Laboratory of Anthropometry, Health Sciences Center (CCS), UFSC, using the Lunar Prodigy Advance equipment, model Discovery WiFan-Beam, serial number 81593, (GE Medical Systems, Madison, Wisconsin, United States). X-ray attenuation in body tissues was computed using the Encore 2004 software, version 8.10.027 (GE Lunar Corporation, Madison, Wisconsin, United States).

Internal quality control was obtained through a daily automatic calibration process that preceded evaluations and which was done in accordance with the manufacturer’s instructions. One researcher who had previously been trained to make these measurements, was responsible for all evaluations and followed all the procedures standardized by the equipment manufacturer. During the evaluations, the participants were instructed to wear appropriate clothing (top or bikini and swimming trunks) and be barefoot, without use of earrings and/or finger rings or any metallic adornments.

The evaluations performed through DXA produced measurements of subtotal BMD (without the head region) and subtotal BMC (without the head region), with corrections for height and body fat percentage. These subtotal measurements (BMD and BMC) are considered to be parameters with good accuracy and reproducibility, compared with total BMD and BMC, which can dilute changes in bone mass.^14^

#### Independent variables

The physical growth variables evaluated were the age/height z score, BMI/age z score and fat percentage z score. Z scores allow specific values to be compared with the population, taking into account typical values and dispersion, and considering age and sex.^15^

To calculate the z score standardized according to age and height, height was obtained using a stadiometer (Altura Exata, Belo Horizonte, Brazil), with measurement capacity from 115 cm to 210 cm and resolution of 0.1 cm, in accordance with the recommendations proposed by the International Society for the Advancement of Kinanthropometry (ISAK).^16^ Chronological age was collected through an interview.

To calculate the z score standardized according to age and BMI, body mass was obtained using a portable digital scale (Tanita; model BF-683W) with 0.1 kg accuracy and capacity for up to 150 kg. BMI was calculated from body mass and height measurements. For body mass measurements, the recommendations of the International Society for the Advancement of Kinanthropometry (ISAK)^16^ were followed.

To calculate the z score standardized according to fat percentage, the fat percentage was obtained through DXA. Test-retest reproducibility for body fat was examined in an independent sample of the present study (n = 10) that was similar in age and sex distribution: 11.6 ± 5.8 and 11.5 ± 5.9 kg; intraclass correlation coefficient (ICC) = 1.00; 95% confidence interval (CI) = 0.99-1.00.^17^

Bone age was assessed by means of left-limb hand-wrist radiography, following standardized procedures,^18^ in which the hand-wrist region of the left limb, in the anteroposterior direction, was positioned at a distance of 100.0 cm from the equipment. The fingers were extended, with the third finger in line with the forearm. The forearm, palm and fingers were in contact with the film plate and the x-ray beam was centered in the distal region of metacarpal III. Bone age was determined by comparing the radiography obtained with a series of standard radiographs that represent the skeletal maturation process in healthy subjects.^18^ The radiographs were read by a radiology specialist and all procedures were performed at the Department of Radiology of HIJG.

#### Covariates

The variables of sex (male or female) and family income were collected using a questionnaire developed for this study. Family income was determined as the number of minimum monthly wages (1 minimum wage at the time of the study was R$ 724.00) and was categorized in the following ranges: ≤ 2 minimum wages; > 2 to 5 minimum wages; or > 5 minimum wages.

Information on HIV infection was obtained through analysis of the medical records, from which information was obtained regarding CD4+ lymphocyte count, viral load and type of ART. The type of ART was categorized as follows: use of ART with protease inhibitors (PI); use of ART without PI; or no use of ART.

Data on moderate to vigorous physical activity (MVPA) and sedentary behavior were collected by means of triaxial Actigraph accelerometers (3.8 x 3.7 x 1.8 cm), model GT3XE-Plus (Manufacturing Technology Inc., Fort Walton Beach, United States). These allowed measurement of acceleration produced through body movement.^19^ This equipment was used continuously over a period of 7 to 14 days, including weekends.^19,20^ The participants were instructed to attach the equipment to the right hemibody at the waistline early in the morning and use it until the end of the day, and only to remove it for activities in water and for sleeping.^20^ The motion sensor was calibrated and the stored information was downloaded to the Actilife 6.0 software (ActiGraph, Pensacola, Florida, United States) in a process that took 15 seconds. The subject’s usual physical activity was represented by at least four valid days, comprising three weekdays and one weekend day containing at least 10 hours of information (600 minutes), after removing nonuse periods of at least 60 consecutive zeros.^21^ Contacts through phone calls and messages, at least once a week, were used to ensure regular use of the device. Verbal and written instructions were provided to subjects and their guardians before the device was used.^20^ For subjects who did not present valid days through previous measurements, up to two further attempts to use the device were made.

The numbers of minutes at different physical activity intensities were proportionally adjusted to a 14-hour period, considering that this is the average time agreed for this population. This was done through the following formula: adjusted minutes = (registered minutes/time of use) * (14 * 60).^22^ The number of minutes of MVPA was obtained considering the cutoff point proposed by Evenson et al.^23^ Uninterrupted blocks (bouts) of at least five and ten minutes of MVPA were derived.^22^ The test-retest reproducibility of this protocol was examined using a subsample of the present study (42.6 ± 23.2 and 34.5 ± 17.2 minutes of MVPA on the first and second visits, respectively; ICC = 0.90; 95% CI = 0.74 to 0.96; n = 17). In view of the recommendations of the World Health Organization regarding MVPA, continuous data were dichotomized into “met recommendations” (≥ 60 minutes of MVPA per day) and “did not meet recommendations” (< 60 minutes of MVPA per day).^24^ Regarding sedentary behavior, the accumulation of time spent with sedentary behavior was ascertained, and this variable was continuously analyzed.

Muscle strength was assessed using a handgrip test. The Saehan hydraulic dynamometer (Model SH5001, Saehan Corporation, Masan, Korea) was used. The device was positioned between the fingers and the palm at the base of the thumb, with extended elbow joint. The opening of the dynamometer was adjusted so that the second joint of the fingers fitted into the dynamometer handle. During the test, the dynamometer or the hand did not touch any other objects. Right handgrip (D) and left handgrip (E) were alternately assessed, with two attempts per assessment. The best left and right scores obtained in each test were added together to obtain the general score (D + E, in kilograms). The standardization of the Canadian Society for Exercise Physiology^25^ was adopted.

### Statistical analysis

Descriptive analysis was firstly performed (mean and standard deviation; and median and interquartile range) and observations with missing data were excluded. Kurtosis and asymmetry were assessed in order to ascertain whether the data had normal distribution (range from -2 to + 2), and histograms were also analyzed to identify data distribution normality. Pearson or Spearman linear correlation and simple and multiple linear regression were used to verify correlations and associations between dependent and independent variables, respectively. For multiple linear regression analysis, three models were built. Hierarchical adjusted analysis was performed,^26^ with division into three blocks: distal (sex and income), intermediate (viral load, CD4 and use of ART) and proximal (MVPA, sedentary behavior and muscle strength). All the variables remained in the adjusted model, regardless of the P-value of the crude analysis, using the forward method.

Regression coefficients (β), 95% confidence intervals and determination coefficients were estimated for each model analyzed (R^2^) and for multicollinearity diagnosis (VIF), and the Cohen’s D effect size was calculated.^27^ For all analyses, the SPSS software (Statistical Package for the Social Sciences - IBM: SPSS Statistics, Chicago, United States), version 23.0, was used. P-values ≤ 0.05 were taken to be statistically significant.

## RESULTS

Sixty-three children and adolescents who had been diagnosed with HIV participated in the study (males = 28; females = 35). In the full sample, the mean age was 12.14 years (± 1.95), the mean height was 147.3 cm (± 13.08), the mean body mass was 39.9 kg (± 11.04) and the mean bone age was 12.02 years (± 2.68) (data not shown in tables/figures). The sample characteristics are shown in Table 1.

**Table 1.** Characteristics of the children and adolescents diagnosed as HIV+ (n = 63)

Total sample (n = 63)		
	Mean (± SD)	Median (p25; p75)
Subtotal BMC/height (kg/cm)	8.07 (3.13)	7.94 (5.54; 9.65)
Subtotal BMD (kg/cm²)	8.37 (11.49)	8.23 (7.52; 9.12)
Age/height z score	-0.53 (1.12)	-0.58 (-1.31; 0.36)
BMI/age z score	-0.19 (1.01)	-0.20 (-0.87; 0.58)
Fat % z score	-1.55 (1.87)	-1.40 (-2.65; -0.08)
Bone age (years)	12.02 (2.68)	12.50 (10.00; 14.00)
Viral load (log)	2.16 (0.97)	1.60 (1.60; 2.63)
CD4+ lymphocyte (cells/mm^3^)	857.63 (367.73)	819.00 (574.50; 1096.00)
Sedentary behavior (min)	388.93 (216.61)	458.54 (214.11; 555.00)
Muscle strength (kg)	21.05 (9.78)	19.00 (14.00; 26.00)
n (%)		
**MVPA**		
Met recommendations	20 (30.80)	
Did not meet recommendations	43 (69.20)	
**ART**		
No use of ART	11 (16.90)	
ART with PI	15 (23.10)	
ART without PI	39 (60.00)	
**Monthly income**		
≤ 2 minimum wages	26 (40.00)	
> 2 to 5 minimum wages	23 (35.40)	
> 5 minimum wages	16 (24.60)	
**Sex**		
Male	28 (46.20)	
Female	35 (53.80)	

HIV = human immunodeficiency virus; BMC = bone mineral content; BMD = bone mineral density; SD = standard deviation; BMI = body mass index; ART = antiretroviral drugs; PI = protease inhibitors; n = sample number; kg = kilograms; cm = centimeters; min = minutes; MVPA = moderate to vigorous physical activity.

Table 2 shows the correlation matrix among all the variables. Subtotal BMC/height showed a significant positive correlation with subtotal BMD, height/age z score, BMI/age z score, fat percentage z score and bone age. Subtotal BMD showed a significant positive correlation with BMI/age z score, fat percentage z score and bone age and showed a negative correlation with CD4+ lymphocytes (Table 2).

**Table 2.** Pearson and Spearman correlation matrix between the variables investigated among children and adolescents diagnosed as HIV+

Total sample (n = 63)													
Pearson and Spearman correlation coefficient													
	MVPA	Viral load	CD4+ lymphocyte	SB	Subtotal BMC/height	Subtotal BMD	Age/height z score	BMI/age z score	Fat % z score	Muscle strength	Bone age	Sex	ART
Viral load (log)	-0.038												
CD4+ lymphocyte	0.125	-0.495^*‡^											
SB	-0.168	0.219	-0.192										
Subtotal BMC/height	0.020	-0.216	-0.217	-0.159									
Subtotal BMD	0.033	-0.093	-0.274^†‡^	-0.119	0.980^*‡^								
Age/height z score	0.122	-0.158	0.201	-0.027	0.240^*‡^	0.141							
BMI/age z score	0.045	-0.294^*‡^	-0.107	0.108	0.407^*‡^	0.397^*‡^	0.283^*‡^						
Fat % z score	-0.092	0.087	-0.350^†‡^	0.415^*‡^	0.232^*‡^	0.242^†‡^	-0.007	0.555^*‡^					
Muscle strength	-0.091	0.037	0.018	0.110	0.038	-0.03	0.014	-0.062	0.03				
Bone age (years)	-0.170	-0.048	-0.235 ^†‡^	-0.006	0.838^*‡^	0.807^*‡^	0.139	0.226	0.197	0.022			
Income	0.125	0.030	-0.067	-0.104	-0179	-0.054	0.120	0.017	0.126	-0.156	-0.161	-0.630	0.069
Sex	0.140	0.850	-0.126	-0.080	-0.020	0.026	0.113	0.104	-0.100	0.274	-0.180		
ART	-0.003	-0.062	0.170	-0.100	0.116	0.134	-0.043	-0.062	0.083	-0.025	0.193		

* P-value < 0.001; ^†^P-value < 0.05. ^‡^Pearson correlation; ^§^Spearman correlation; % = percentage. HIV = human immunodeficiency virus; MVPA = moderate to vigorous physical activity; SB = sedentary behavior; BMC = bone mineral content; BMD = bone mineral density; ART = antiretroviral drugs; BMI = body mass index.

Simple linear regression analysis between subtotal BMD and physical growth among these children and adolescents diagnosed with HIV demonstrated that subtotal BMD was directly associated with bone age, which explained 64% of BMD variability (P < 0.001). After adding the covariates of sex, family income, viral load, CD4+ lymphocytes, ART, MVPA, sedentary behavior and muscle strength, subtotal BMD was found to be directly associated with bone age and the model explained 79% of the subtotal BMD variability (P < 0.001) (Table 3).

**Table 3.** Simple and multiple linear regression between subtotal BMD and physical growth among children and adolescents diagnosed as HIV+

Total sample (n = 63)						
	β (95% CI)	β st	R²	P	VIF	Cohen’s D
**Bone age**						
Crude model	0.03 (0.02; 0.04)	0.80	0.64	**< 0.01**	–	1.77
Model I	0.03 (0.02; 0.40)	0.80	0.66	**< 0.01**	1.01	1.94
Model II	0.03 (0.02; 0.39)	0.78	0.70	**< 0.01**	1.13	2.33
Model III	0.03 (0.02; 0.38)	0.79	0.79	**< 0.01**	1.38	3.76
**Age/height z score**						
Crude model	0.14 (-0.11; 0.40)	0.14	0.02	0.26	–	0.02
Model I	0.01 (-0.01; 0.45)	0.18	0.04	0.15	1.03	0.04
Model II	0.01 (0.01; 0.52)	0.26	0.04	**0.02**	1.03	0.04
Model III	0.00 (-0.32; 0.38)	0.06	0.22	0.63	1.15	0.28
**BMI/age z score**						
Crude model	0.04 (0.01; 0.71)	0.39	0.14	**< 0.01**	–	0.16
Model I	0.04 (0.02; 0.75)	0.42	0.18	**< 0.01**	1.01	0.21
Model II	0.03 (0.01; 0.65)	0.32	0.29	**< 0.01**	1.02	0.47
Model III	0.03 (0.01; 0.63)	0.29	0.47	**< 0.01**	1.25	0.88
**Fat % z score**						
Crude model	0.01 (0.00; 0.30)	0.24	0.04	**0.05**	–	0.04
Model I	0.01 (0.01; 0.30)	0.26	0.07	**0.04**	1.04	0.07
Model II	0.08 (-0.01;0.02)	0.12	0.23	0.34	1.24	0.29
Model III	0.02 (0.01; 0.40)	0.44	0.36	**< 0.01**	1.69	0.56

BMD = bone mineral density; HIV = human immunodeficiency virus; CI = confidence interval; st = standardized; VIF = multicollinearity diagnosis; BMI = body mass index. Model I: sex and income; Model II: sex, income, viral load, lymphocyte CD4+ and use of antiretroviral; Model III: sex, income, viral load, lymphocyte CD4+, use of antiretroviral, moderate-vigorous physical activity, sedentary behavior and muscle strength.

Subtotal BMD was directly associated with BMI/age z score, which explained 14% of subtotal BMD variability (P < 0.001) in the crude model. After adding the covariates of sex, family income, viral load, CD4, use of ART, MVPA, sedentary behavior and muscle strength (model III), subtotal BMD remained directly associated with BMI/age z score and the model explained 47% of subtotal BMD variability (P < 0.001). Subtotal BMD was directly associated with fat percentage z score, which explained 4% of its variability (p = 0.005) in the crude model. The variables of sex, income, viral load, CD4+ lymphocytes, use of ART, MVPA, sedentary behavior and muscle strength (model III) were directly associated with fat percentage z score, which explained 36% of subtotal BMD variability (P < 0.001) (Table 3).

Simple linear regression analysis demonstrated that subtotal BMC/height was directly associated with bone age, which explained 69% of BMC variability (P < 0.001). The variables of sex, income, viral load, CD4+ lymphocytes, ART, MVPA, sedentary behavior and muscle strength (model III) were directly associated with bone age, which explained 76% of BMC variability (P < 0.001). Subtotal BMC/height was directly associated with height/age z score, which explained 3% of BMD variability (P = 0.005) in the crude model. The variables of sex, income, viral load, CD4+ lymphocytes, use of ART, MVPA, sedentary behavior and muscle strength (model III) were directly associated with height/age z score, which explained 28% of subtotal BMC/height variability (P = 0.004). Subtotal BMC/height was directly associated with BMI/age z score, which explained 16% of subtotal BMC/height variability in the crude model (P < 0.001). The covariates of sex, family income, viral load, CD4+ lymphocytes, use of ART, MVPA, sedentary behavior and muscle strength (model III) were associated with BMI/age z score, which explained 77% of subtotal BMC/height variability (P < 0.001). Subtotal BMC/height was not associated with fat percentage z score in the simple analysis. The variables of sex, income, viral load, CD4+ lymphocytes, use of ART, MVPA, sedentary behavior and muscle strength (model III) were directly associated with fat percentage z score, which explained 52% of subtotal BMC/height variability (P < 0.001) (Table 4).

**Table 4.** Simple and multiple linear regression between subtotal BMC/height and physical growth among children and adolescents diagnosed as HIV+

Total sample (n = 63)						
	β (95% CI)	β st	R²	P	VIF	Cohen’s D
**Bone age**						
Crude model	0.96 (0.79; 1.12)	0.83	0.69	**< 0.01**	–	2.22
Model I	0.95 (0.79; 1.12)	0.83	0.70	**< 0.01**	1.01	2.33
Model II	0.82 (0.49; 1.14)	0.71	0.71	**< 0.01**	3.42	2.44
Model III	0.86 (0.56; 1.16)	0.75	0.76	**< 0.01**	4.40	3.16
**Age/height z score**						
Crude model	0.94 (0.41; 1.41)	0.85	0.03	**0.05**	–	0.03
Model I	0.67 (0.13; 1.35)	0.26	0.04	**0.05**	1.00	0.04
Model II	0.74 (0.02; 1.46)	0.26	0.04	**0.04**	1.03	0.04
Model III	0.95 (0.32; 1.59)	0.34	0.28	**0.04**	1.10	0.38
**BMI/age z score**						
Crude model	1.26 (0.54;1.97)	0.40	0.16	**< 0.01**	–	0.19
Model I	1.26 (0.54; 1.97)	0.40	0.17	**< 0.01**	1.01	0.20
Model II	1.04 (0.60;1.49)	0.33	0.74	**< 0.01**	1.11	2.84
Model III	1.24 (0.77; 1.73)	0.40	0.77	**< 0.01**	1.04	3.34
**Fat % z score**						
Crude model	0.38 (-0.02; 0.79)	0.23	0.03	0.06	–	0.03
Model I	0.43 (0.01; 0.86)	0.26	0.07	**0.04**	1.04	0.07
Model II	0.21 (-0.21; 0.64)	0.12	0.18	0.32	1.24	0.21
Model III	0.67 (0.78; 19.54)	0.39	0.52	**< 0.01**	1.01	1.08

BMC = bone mineral content; HIV = human immunodeficiency virus; CI: confidence interval; st = standardized; VIF: multicollinearity diagnosis; Model I: sex and income, Model II: sex, income, viral load, lymphocyte CD4+ and use of antiretroviral; Model III: sex, income, viral load, lymphocyte CD4+, use of antiretroviral, moderate-vigorous physical activity, sedentary behavior and muscle strength.

## DISCUSSION

The main findings of this study were: 1) there were direct associations between subtotal BMD and bone age, BMI/age z score and fat percentage z score, even after adjusting for covariates; and 2) subtotal BMC/height was directly associated with bone age, height/age z score, BMI/age z score and fat percentage z score even after adjusting for covariates.

In the present study, a direct association between subtotal BMD and bone age was observed. A previous study carried out among 1,218 children aged 6-18 years of both sexes, without any HIV diagnosis, also found a direct correlation between BMD and bone age.^28^ One possible explanation for the association between BMD and bone age may be the correlation between the beginning, peak and end of the puberty growth spurt and specific bone development states.^10^ The period from the beginning to the end of the puberty growth spurt lasts approximately two years, and the peak growth rate occurs around one year after the start of the growth spurt.^29^ Linear growth in adolescence is greatest during pubertal development, with no net gain in bone mass after peak bone mass is reached.^30^ Thus, peak bone mass is considered to form the bone reservoir for future life and is the factor that determines BMD in adulthood and, consequently, the risk of osteoporotic fractures.^30^ It has thus been observed that linear growth is closely linked to skeletal development. However, because HIV infection and ART can affect bone development in children and adolescents diagnosed with HIV, the relationship between BMD and bone age is not fully understood in this population.^31^

Furthermore, a study among male adolescents diagnosed with HIV infection, in comparison with a control group, showed that there were similar values for BMC and BMD in the pre-pubertal maturation stage in the two groups. In the pubertal and post-pubertal maturational stage group, BMC and BMD values were lower in children and adolescents diagnosed with HIV infection, thus demonstrating that the differences in BMC and BMD become more evident with the advancement of puberty.^32^

In the present study, direct associations between subtotal BMD and BMI/age z score and fat percentage z score were found. In other studies on pediatric populations diagnosed with HIV, results similar to those found in the present study were reported.^6,9^ The possible explanation for these findings is the fact that fat cells, adipocytes and bone formation cells (osteoblasts) all originate in mesenchymal stem cells, a heterogeneous group of multipotent non-hematopoietic stromal cells that are capable of becoming differentiated into mesodermal and non-mesodermal cells (adipocytes and osteoblasts).^33^ Thus, the direct association between total body fat and BMD identified in the present study can be explained by the common cellular matrix from which both adipocytes and osteoblasts originate.^34^ In addition, body fat positively contributes to leptin secretion,^35^ which is directly related to greater proliferation and differentiation of osteoblasts and osteoclasts, which provides balance in the remodeling process of bone structures and BMD maintenance.^35^

Subtotal BMC/height was directly associated with bone age, height/age z score, BMI/age z score and fat percentage z score in the children and adolescents diagnosed with HIV of the present study. Previous studies that investigated associations between bone development and physical growth parameters only assessed BMD, with no data regarding BMC.^6,9,10^ This fact limits comparisons of the findings from the present study with previous data in the literature. A study carried out among prepubertal children diagnosed with HIV infection showed lower values for total BMC than those of their peers without a diagnosis of HIV infection, when considering age and body mass in the analyses.^36^ BMC assessment among children and adolescents diagnosed with HIV is important, given that it is during childhood and adolescence that both bone deposition and bone mass formation exceed resorption, thus implying increases in BMC and BMD at phases that coincide with accelerated weight and height growth.^37^ In addition, BMC assists in bone modeling regulation.^38^

In a study that compared children and adolescents diagnosed with HIV with children and adolescents without an HIV diagnosis, it was reported that exposure to HIV and use of ART since birth were associated with lower bone mass.^7^ In children and adolescents diagnosed with HIV, lower bone concentrations and mineralization rates may occur due to impaired adequate physical development and subsequently delayed puberty, which can be related to HIV infection.^3,4^

The present study had limitations that need to be considered when interpreting its data: the cross-sectional design did not allow inferences regarding cause-effect relationships. The sample used, which had a wide age range that included pre and post-pubertal children and adolescents, can also be considered to have been a limitation: because of the sample size, it was not possible to perform stratifications according to maturation stage.

Some strengths of this study include its use of highly reliable methods, such as DXA to evaluate BMD and BMC, accelerometers to measure MVPA and sedentary behavior and hand-wrist radiography analyzed by a radiologist to obtain bone age.

## CONCLUSIONS

Through the findings of the present study, it could be concluded that subtotal BMD and subtotal BMC/height were directly associated with physical growth indicators (BMD: bone age, BMI/age z score and fat percentage z score; and BMC: bone age, height/age z score, BMI/age z score and fat percentage z score, respectively), even after adjusting for sex, family income, viral load, CD4+ lymphocytes, use of ART, MVPA, behavior sedentary and muscle strength. Assessing and improving bone health in children and adolescents diagnosed with HIV is crucial, in order to minimize the risk of bone complications over the long term. It needs to be emphasized that with prolonged use of ART, there may be significant bone mass reductions. Thus, monitoring of physical growth indicators that are simple to assess, such as age/height z score, BMI/age z score and fat percentage z score can be useful for identifying possible bone mass deficits in children and adolescents diagnosed with HIV.
